# Influences of remote ischemic preconditioning on postoperative delirium and cognitive dysfunction in adults after cardiac surgery: a meta-analysis of randomized controlled trials

**DOI:** 10.1186/s13741-021-00216-1

**Published:** 2021-12-10

**Authors:** Yuchen Jing, Bai Gao, Xi Li

**Affiliations:** 1 Department of Vascular Surgery, The First Affiliated Hospital of China Medical University, No. 155 Nanjing Bei Street, Heping District, Shenyang, 110001 China; 2 Department of Neurology, Shengjing Hospital Affiliated to China Medical University, Shenyang, 110004 China

**Keywords:** Remote ischemic preconditioning, Postoperative delirium, Postoperative cognitive dysfunction, Cardiac surgery, Meta-analysis

## Abstract

**Background:**

Remote ischemic preconditioning (RIPC) has been suggested to confer neuroprotective effect. However, influences of RIPC on postoperative delirium (POD) and cognitive dysfunction (POCD) in adults after cardiac surgery are less known. We performed a meta-analysis of randomized controlled trials (RCTs) to evaluate the effects of RIPC on POD and POCD.

**Methods:**

Relevant studies were obtained by search of PubMed, Embase, and Cochrane’s Library databases. A random-effect model was used to pool the results.

**Results:**

Ten RCTs including 2303 adults who received cardiac surgery were included. Pooled results showed that RIPC did not significantly affect the incidence of POD (six RCTs, odds ratio [OR] 1.07, 95% confidence interval [CI] 0.81 to 1.40, *P* = 0.65) with no significant heterogeneity (*I*^2^ = 0%). In addition, combined results showed that RIPC did not significantly reduce the incidence of POCD either (six RCTs, OR 0.64, 95% CI 0.37 to 1.11, *P* = 0.11) with moderate heterogeneity (*I*^2^ = 44%). Sensitivity analysis by excluding one RCT at a time showed consistent results (*P* values all > 0.05).

**Conclusions:**

Current evidence from RCTs did not support that RIPC could prevent the incidence of POD or POCD in adults after cardiac surgery. Although these findings may be validated in large-scale RCTs, particularly for the results of POCD, based on these findings, RIPC should not be routinely used as a preventative measure for POD and POCD in adult patients after cardiac surgery.

## Introduction

Postoperative delirium (POD) and postoperative cognitive dysfunction (POCD) are common postoperative cognitive disorders in patients following cardiac surgery with general anesthesia (Thiele et al., 2021; Kapoor, 2020). Clinically, POD is defined as a transient disturbance of the consciousness, attention, cognition, and perception of the patient, which could affect up to 50% of elderly patients after cardiac surgery such as coronary artery bypass grafting (CABG) (Duning et al., 2021; Sanson et al., 2018). As for POCD, it is typically presented as a decline in cognitive function after the surgery, which could affect both the young and old patients after cardiac surgery (Hua & Min, 2020; Glumac et al., 2019). Previous studies have confirmed that both POD and POCD are associated with prolonged hospitalization, impaired functional ability, and increased mortality in patients after cardiac surgery (Goldberg et al., 2020; Labaste et al., 2020; Brown et al., 2018; Li et al., 2021). Therefore, development of a novel strategy to prevent the incidence of POD/POCD is of importance in clinical practice. Remote ischemic preconditioning (RIPC) refers to a strategy which confers protective efficacy to target organs by inducing short episodes of ischemia and reperfusion in distant tissue (Heusch et al., 2015; Pickard et al., 2015). Emerging evidence has indicated that RIPC during the perioperative period may reduce the incidence of postoperative complications in patients after cardiac surgery, such as acute kidney injury (Liu et al., 2021) and myocardial injury (Moscarelli et al., 2021). Interestingly, recent evidence shows that RIPC may be effective for slowing cognitive decline in patients with cerebral small-vessel disease (Wang et al., 2017), subcortical ischemic vascular dementia (Liao et al., 2019), and after ischemic stroke (Landman et al., 2019). However, previous studies have not fully determined whether RIPC is effective in reducing POD/POCD in patients after cardiac surgery (Jing & Zheng, 2011; Joung et al., 2013; Meybohm et al., 2013; Hudetz et al., 2015; Meybohm et al., 2015; Brown, 2016; Kim et al., 2017; Meybohm et al., 2018; Gasparovic et al., 2019; Wang et al., 2019). Accordingly, we performed a meta-analysis of randomized controlled trials (RCTs) to systematically evaluate the potential influences of RIPC on postoperative cognitive complications in patients following cardiac surgery.

## Methods

The PRISMA (Preferred Reporting Items for Systematic Reviews and Meta-Analyses) statement (Moher et al., 2009) and the Cochrane Handbook guidelines (Higgins & Green, 2011) were followed during the designing and implementation of the study.

### Search strategy

PubMed, Embase, and the Cochrane Library (Cochrane Center Register of Controlled Trials) databases were searched for relevant studies with a combined strategy of (1) “ischemic preconditioning” OR “remote ischemic preconditioning” OR “RIPC”, (2) “cardiac surgery” OR “heart surgery” OR “postoperative” OR “cognition” OR “cognitive” OR “delirium” OR “dementia”, and (3) “random” OR “randomized” OR “randomized” OR “randomly.” Only clinical studies were considered. The references of related reviews and original articles were also searched as a complementation. The final database search was conducted on April 20, 2021.

### Study selection

Studies that fulfilled the following criteria were included as follows: (1) articles published in English or Chinese, (2) designed as parallel-group RCTs, (3) included adult patients scheduled for open heart surgery who were randomly allocated to a RIPC treatment group or a control group, and (4) reported the incidence of POD and/or POCD in the perioperative periods. The diagnostic criteria of POD and POCD outcomes in the meta-analysis were in accordance with that applied in the included studies. Reviews, studies with children or neonates, studies of non-cardiac surgery, preclinical studies, observational studies, and repeated reports were excluded.

### Data extraction and quality assessment

Database search, data extraction, and quality evaluation were conducted by two independent authors. If disagreement occurred, it was resolved by discussion with the corresponding author. We extracted data regarding study information (first author, publication year, and study country), study design (blind or open-label), patient information (number of participants, mean age, and sex), surgery type, perioperative anesthetics, and anesthesia depth monitoring, RIPC protocol, and diagnostic strategy for patients with POD and/or POCD. Quality evaluation was achieved using the Cochrane’s Risk of Bias Tool (Higgins & Green, 2011) according to the following aspects: (1) random sequence generation, (2) allocation concealment, (3) blinding of participants and personnel, (4) blinding of outcome assessors, (5) incomplete outcome data, (6) selective outcome reporting, and (7) other potential bias.

### Statistical analysis

Incidence of POD and POCD was separately evaluated via odds ratios (ORs) and their 95% confidence intervals (CIs) in this meta-analysis. We used the Cochrane’s Q test to detect the heterogeneity (Higgins & Thompson, 2002). The *I*^2^ statistic was also calculated, and an *I*^2^ > 50% reflected significant heterogeneity. Pooled analyses were calculated using a random-effect model because this method incorporates the influence of potential heterogeneity and retrieves a more generalized result (Higgins & Green, 2011). Sensitivity analysis by excluding one study at a time was used to evaluate the influence of each study on the pooled results of the meta-analysis (Higgins & Green, 2011). Publication bias was evaluated by visual inspection of funnel plots, and the Egger’s regression asymmetry test (Egger et al., 1997). If high risk of publication bias was suggested, a “trim-and-fill” analysis was used for further evaluation, which estimates the influence of possible studies with negative findings on the meta-analysis outcome (Higgins & Green, 2011). *P* values < 0.05 were considered statistically significant. The RevMan (Version 5.1; Cochrane, Oxford, UK) and Stata software (Version 12.0; Stata, College Station, TX) were applied for statistical analyses.

## Results

### Search results

**Fig. 1:**
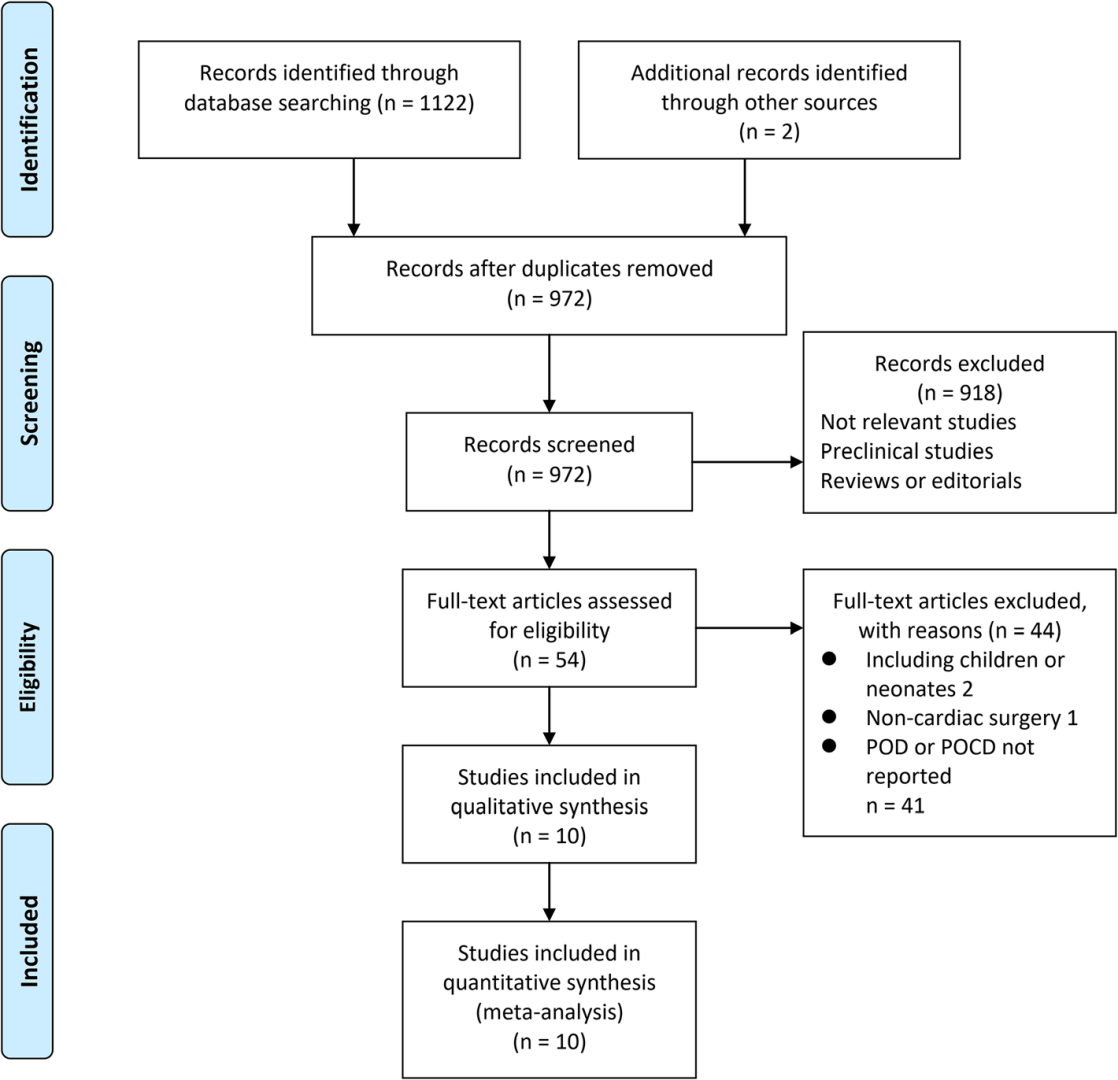
Flowchart of literature search

The process of database search and study identification was shown in Fig. 1. Briefly, 1124 articles were obtained through the database search, and 972 were retrieved after exclusion of duplicated records. Among them, 918 articles were subsequently excluded based on titles and abstracts primarily because these studies were irrelevant to the aim of the meta-analysis. Of the 54 articles that underwent full-text review, 44 were further excluded for the reasons presented in Fig. 1 Finally, 10 RCTs (Jing & Zheng, 2011; Joung et al., 2013; Meybohm et al., 2013; Hudetz et al., 2015; Meybohm et al., 2015; Brown, 2016; Kim et al., 2017; Meybohm et al., 2018; Gasparovic et al., 2019; Wang et al., 2019) were included.

### Study characteristics

Table 1 shows the characteristics of the included studies. Overall, 10 RCTs with 2303 patients were included in the current meta-analysis (Jing & Zheng, 2011; Joung et al., 2013; Meybohm et al., 2013; Hudetz et al., 2015; Meybohm et al., 2015; Brown, 2016; Kim et al., 2017; Meybohm et al., 2018; Gasparovic et al., 2019; Wang et al., 2019). These studies were published between 2011 and 2019 and performed in China (Jing & Zheng, 2011; Wang et al., 2019), Korea (Joung et al., 2013; Kim et al., 2017), Germany (Meybohm et al., 2013; Meybohm et al., 2015; Meybohm et al., 2018), and the USA (Hudetz et al., 2015; Brown, 2016; Gasparovic et al., 2019), respectively. All of these studies were double-blinded RCTs. Eight studies included patients with on-pump heart surgeries (Jing & Zheng, 2011; Meybohm et al., 2013; Hudetz et al., 2015; Meybohm et al., 2015; Brown, 2016; Kim et al., 2017; Meybohm et al., 2018; Gasparovic et al., 2019), while the remaining two included patients with off-pump surgeries (Joung et al., 2013; Wang et al., 2019). General anesthesia with intravenous anesthetics was applied in the included studies. In nine studies, RIPC was performed after anesthesia induction (acute RIPC) (Jing & Zheng, 2011; Joung et al., 2013; Meybohm et al., 2013; Hudetz et al., 2015; Meybohm et al., 2015; Brown, 2016; Meybohm et al., 2018; Gasparovic et al., 2019; Wang et al., 2019), while in one study RIPC was performed 24~48h before the surgery (chronic RIPC) (Kim et al., 2017). The protocol of RIPC included 3~4 cycles of upper or lower limb ischemia (5 min of blood pressure cuff inflation to a pressure of 200 mmHg or at least a pressure that was 40 mmHg higher than the systolic arterial pressure), followed by 5~10 min reperfusion (with the cuff deflated). Uninflated cuffs were used on patients in the control group after anesthesia for studies evaluating the acute effect of RIPC (Jing & Zheng, 2011; Joung et al., 2013; Meybohm et al., 2013; Hudetz et al., 2015; Meybohm et al., 2015; Brown, 2016; Meybohm et al., 2018; Gasparovic et al., 2019; Wang et al., 2019), while cuff inflated with 10mmHg pressure was applied for patients of control group in the only delayed-effect study (Kim et al., 2017). The outcome of POD was reported in six RCTs (Hudetz et al., 2015; Meybohm et al., 2015; Brown, 2016; Kim et al., 2017; Gasparovic et al., 2019; Wang et al., 2019), which were diagnosed based on instruments of CAM-ICU (Meybohm et al., 2015; Brown, 2016; Kim et al., 2017; Gasparovic et al., 2019; Wang et al., 2019) or ICDSC score (Hudetz et al., 2015). The incidence of POCD events was also reported in six RCTs (Jing & Zheng, 2011; Joung et al., 2013; Meybohm et al., 2013; Hudetz et al., 2015; Meybohm et al., 2018; Gasparovic et al., 2019), most of which were diagnosed by the standard deviation (SD) criteria (Jing & Zheng, 2011; Meybohm et al., 2013; Hudetz et al., 2015; Meybohm et al., 2018; Gasparovic et al., 2019). Specifically, POCD was defined as postoperative performance deterioration by ≥ 1 SD on ≥ 2 tests in four studies (Jing & Zheng, 2011; Meybohm et al., 2013; Meybohm et al., 2018; Gasparovic et al., 2019), by > 20% on ≥ 2 cognitive tests in one study (Joung et al., 2013), and by ≥ 1 SD on ≥ 1 cognitive test in the other study (Hudetz et al., 2015). Patients with POD and POCD were identified within 5~7 days after surgery in all of the included studies.

**Table 1:** Characteristics of the included RCTs

Study	Country	Design	Surgical procedure	No. of patients	Mean age years	Male %	Anesthesia regimen	Anesthesia depth monitoring	Protocols of RIPC	Control	Diagnosis of outcomes
Jing 2011	China	R, DB	On-pump valvular surgery	40	49.5	37.5	Midazolam, fentanyl, rocuronium, sevoflurane;	BIS: 40~60	UL, 260mmHg, 5min × 4, after anesthesia induction and before CPB	Uninflated cuff	POCD: postoperative performance deteriorated by ≥ 1 SD on ≥ 2 tests in MMSE or MoCA
Joung 2013	Korea	R, DB	Off-pump CABG	70	60.0	81.4	Etomidate, propofol, rocuronium, remifentanil;	BIS: 40~60	UL, 200mmHg, 5min × 4, before coronary artery anastomosis	Uninflated cuff	POCD: decreased postoperative test values of > 20% from the baseline in ≥ 2 tests of the 6 cognitive function tests recommended by STS
Meybohm 2013	Germany	R, DB	On-pump heart surgery	180	69.0	81.2	Propofol, rocuronium, sufentanil;	NR	UL, 200mmHg, 5min × 4, after anesthesia induction and before CPB	Uninflated cuff	POCD: postoperative performance deteriorated by ≥ 1 SD on ≥ 2 tests in core battery of 10 tests recommended by STS
Hudetz 2015	USA	R, DB	On-pump heart surgery	30	65.5	100	Midazolam, fentanyl, rocuronium, etomidate, isoflurane;	NR	UL, 200mmHg, 5min × 4, after anesthesia induction and before CPB	Uninflated cuff	POD: ICDSC score based on DSM-IV criteria; POCD: incidence of ≥ 1-SD decline in a brief neuropsychometric battery
Meybohm 2015	Germany	R, DB	On-pump heart surgery	1385	65.9	74.2	Intravenous anesthesia with no volatile anesthetic agents	NR	UL, 200mmHg, 5min × 4, after anesthesia induction and before CPB	Uninflated cuff	POD: CAM-ICU score
Brown 2016	USA	R, DB	On-pump heart surgery	34	74.1	58.8	Midazolam based intravenous anesthesia	NR	UL, 200mmHg, 5min × 3, after anesthesia induction and before CPB	Uninflated cuff	POD: CAM-ICU score
Kim 2017	Korea	R, DB	On-pump heart surgery	160	62.3	53.1	Midazolam, sufentanil, vecuronium, propofol;	BIS: 40~60	UL, 200mmHg, 5min × 4 24~48 hours before surgery	Cuff inflated with 10mmHg pressure	POD: CAM-ICU score
Meybohm 2018	Germany	R, DB	On-pump heart surgery	273	NR	NR	Intravenous anesthesia with no volatile anesthetic agents	NR	UL, 200mmHg, 5min × 4, after anesthesia induction and before CPB	Uninflated cuff	POCD: postoperative performance deteriorated ≥ 1 SD on ≥ 2 tests in core battery of 10 tests recommended by STS
Wang 2019	China	R, DB	Off-pump CABG	65	60.5	73.5	Midazolam, fentanyl, rocuronium, sevoflurane;	BIS: 40~60	UL, SBP+40mmHg, 5min × 4, before surgical incision	Uninflated cuff	POD: CAM-ICU score
Gasparovic 2019	USA	R, DB	On-pump CABG	66	62.0	82.0	Midazolam, sufentanil, rocuronium, sevoflurane;	NR	UL, 200mmHg, 5min × 3, after anesthesia induction and before CPB	Uninflated cuff	POD: CAM-ICU score; POCD: a decrease of minimally ≥ 1 SD in ≥ 2 postoperative neurocognitive tests by MoCA;

*RCTs* randomized controlled trials, *RIPC* remote ischemic preconditioning, *R* randomized, *DB* double-blind, *CABG* coronary artery bypass grafting; Bispectral index, *NR* not reported, *UL* upper limb, *CPB* cardiopulmonary bypass, *POCD* postoperative cognitive dysfunction, *POD* postoperative delirium, *SD* standard deviation, *MMSE* Mini-mental State Examination, *MoCA* Montreal Cognitive Assessment, *STS* Society of Thoracic Surgeons, *DSM-IV* the Diagnostic and Statistical Manual-IV, *CAM-ICU* the Confusion Assessment Method for the Intensive Care Unit, *ICDSC* the Intensive Care Delirium Screening Checklist

### Data quality

Table 2 shows the details of study quality evaluation. All of the included RCTs were double-blind studies. Methods of random sequence generation were reported in seven RCTs (Jing & Zheng, 2011; Joung et al., 2013; Meybohm et al., 2015; Brown, 2016; Meybohm et al., 2018; Gasparovic et al., 2019; Wang et al., 2019), and information of allocation concealment was reported in six RCTs (Meybohm et al., 2013; Hudetz et al., 2015; Meybohm et al., 2015; Kim et al., 2017; Meybohm et al., 2018; Gasparovic et al., 2019). The overall quality score varied between 5 and 7, indicating generally good study quality.

**Table 2:** Details of quality evaluation for the included RCTs according to the Cochrane’s Risk of Bias Tool

Study	Random sequence generation	Allocation concealment	Blinding of participants	Blinding of outcome assessment	Incomplete outcome data addressed	Selective reporting	Other sources of bias	Total
Jing 2011	Low	Unclear	Low	Low	Low	Low	Low	6
Joung 2013	Low	Unclear	Low	Low	Low	Low	Low	6
Meybohm 2013	Unclear	Low	Low	Low	Low	Low	Low	6
Hudetz 2015	Unclear	Low	Low	Low	Low	Low	Low	6
Meybohm 2015	Low	Low	Low	Low	Low	Low	Low	7
Brown 2016	Unclear	Unclear	Low	Low	Low	Low	Low	5
Kim 2017	Low	Low	Low	Low	Low	Low	Low	7
Meybohm 2018	Low	Low	Low	Low	Low	Low	Low	7
Wang 2019	Low	Unclear	Low	Low	Low	Low	Low	6
Gasparovic 2019	Low	Low	Low	Low	Low	Low	Low	7

### Meta-analysis results

**Fig. 2:**
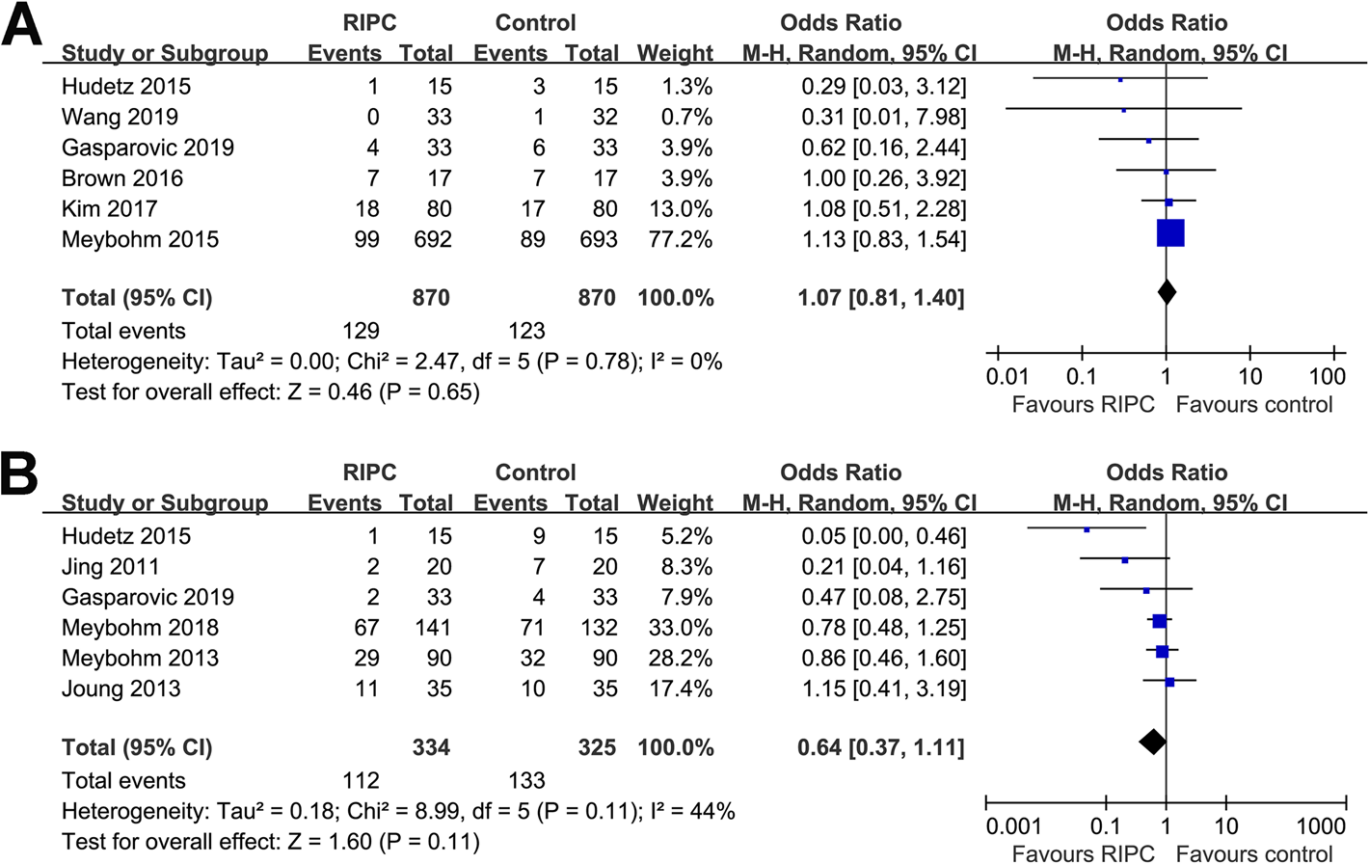
Forest plots for the meta-analysis of effects of RIPC on POD and POCD after cardiac surgery. **A** Forest plots for the meta-analysis of RIPC on POD and **B** forest plots for the meta-analysis of RIPC on POCD

Pooled results showed that RIPC did not significantly affect the incidence of POD (six RCTs, OR 1.07, 95% CI 0.81 to 1.40, *P* = 0.65; Fig. 2A) with no significant heterogeneity (*P* for Cochrane’s *Q* test = 0.78, *I*^2^ = 0%). Sensitivity analysis by excluding one study at a time showed consistent results (Table 3). In addition, combined results showed that RIPC did not significantly reduce the incidence of POCD either (six RCTs, OR 0.64, 95% CI 0.37 to 1.11, *P* = 0.11; Fig. 2B) with moderate heterogeneity (*P* for Cochrane’s *Q* test = 0.11, *I*^2^ = 44%). Sensitivity analysis by omitting one study at a time also did not significantly affect the results (Table 3). Of note, the heterogeneity among the included RCTs for the meta-analysis of POCD was substantially reduced (*I*^2^ = 0%) after excluding the study by Hudetz 2015 (Hudetz et al., 2015), suggesting this study was the major contributor to the heterogeneity.

**Table 3:** Sensitivity analyses

Study excluded	OR (95% CI)	*I*^2^ (%)	*P* for Cochrane’s *Q* test	*P* for overall effect
Influence of RIPC on POD				
Hudetz 2015	1.08 [0.83, 1.42]	0	0.86	0.56
Meybohm 2015	0.86 [0.49, 1.52]	0	0.77	0.61
Brown 2016	1.07 [0.81, 1.41]	0	0.65	0.64
Kim 2017	1.06 [0.80, 1.42]	0	0.65	0.68
Wang 2019	1.07 [0.82, 1.41]	0	0.75	0.60
Gasparovic 2019	1.09 [0.83, 1.43]	0	0.76	0.55
Influence of RIPC on POCD				
Jing 2011	0.72 [0.42, 1.23]	41	0.15	0.23
Joung 2013	0.54 [0.28, 1.04]	51	0.09	0.07
Meybohm 2013	0.50 [0.22, 1.13]	54	0.07	0.10
Hudetz 2015	0.78 [0.56, 1.10]	0	0.52	0.15
Meybohm 2018	0.50 [0.21, 1.19]	55	0.06	0.12
Gasparovic 2019	0.64 [0.34, 1.18]	54	0.07	0.15

*OR* odds ratio, *CI* confidence interval, *POD* postoperative delirium, *POCD* postoperative cognitive dysfunction

### Publication bias

**Fig. 3:**
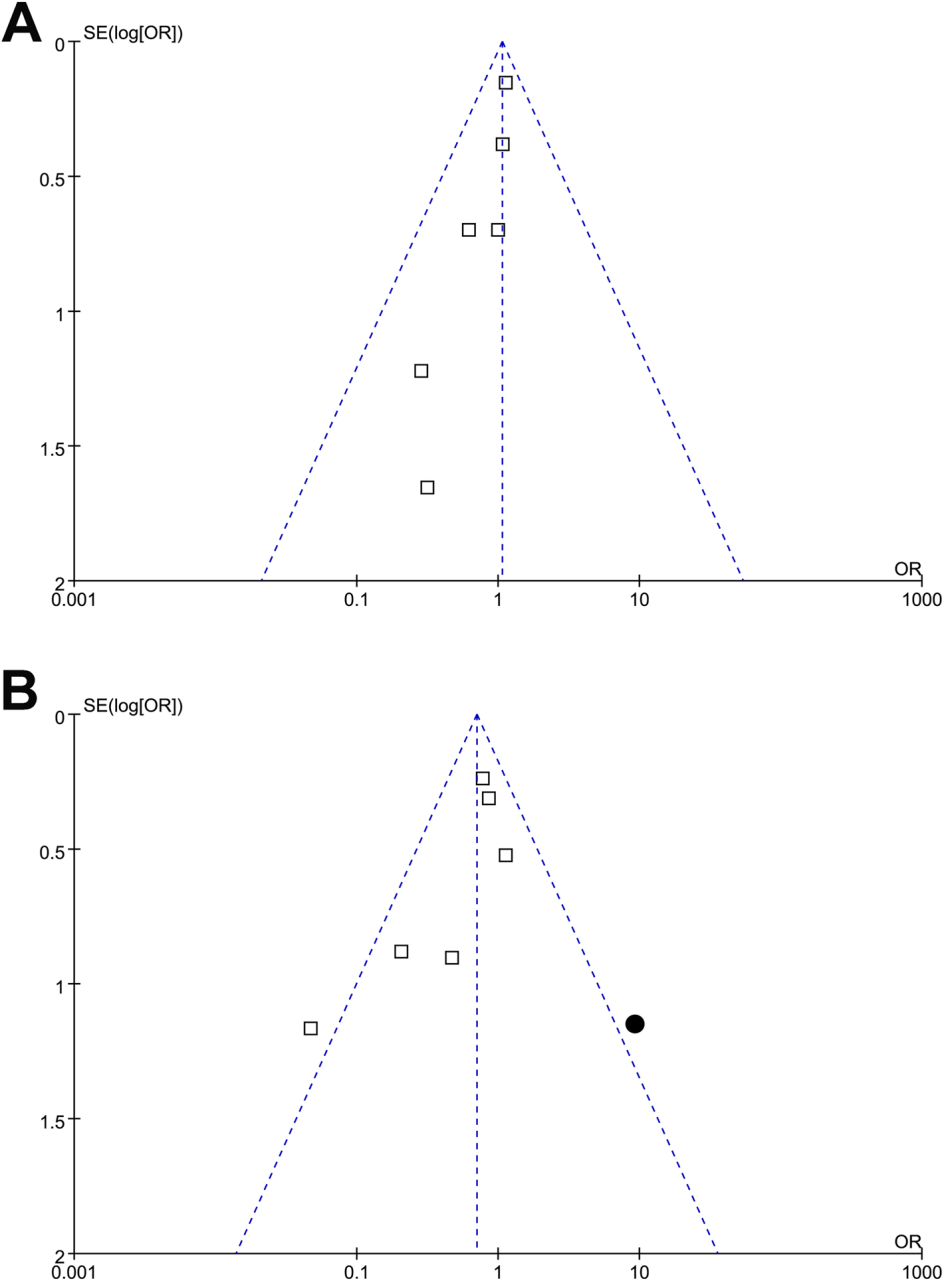
Funnel plots for the effects of RIPC on POD and POCD after cardiac surgery. **A** Funnel plots for the effect of RIPC on POD and **B** funnel plots with “trim-and-fill” analysis for the effect of RIPC on POCD (black circle indicates the hypothesized study to achieve the symmetry of the funnel plots)

The funnel plots for the meta-analysis of POD were symmetrical, suggesting low-risk of publication bias (Fig. 3A). The funnel plots for the meta-analysis of POCD were asymmetrical on visual inspection, suggesting the potential risk of publication bias (Fig. 3B). Egger’s regression tests were not performed since only six RCTs were available for each outcome. We therefore performed a trim-and-fill analysis for the outcome of POCD. As shown in Fig. 3B, incorporating the hypothesized study (black circle) achieved symmetry of the funnel plots, and the results of the meta-analysis remained consistent after including this study (OR 0.72, 95% CI 0.36 to 1.43, *P* = 0.35; *I*^2^ = 61%).

## Discussion

In this study, by pooling the results of available RCTs, the results of the meta-analysis showed that RIPC does not significantly reduce the incidence of POD or POCD in adults following cardiac surgery. To the best of our knowledge, this is the first meta-analysis which summarized the current knowledge regarding the influence of RIPC on postoperative cognitive complications in adults after cardiac surgery. Based on these findings, RIPC should not be routinely used as a preventative measure for POD and POCD in adult patients after cardiac surgery.

For the meta-analysis evaluating the potential effect of RIPC on POD, six RCTs were included (Hudetz et al., 2015; Meybohm et al., 2015; Brown, 2016; Kim et al., 2017; Gasparovic et al., 2019; Wang et al., 2019). Although one of the largest RCT primarily contributed to the results of the meta-analysis (Meybohm et al., 2015), results of the other small-scale RCTs were all consistent, leading to a very low heterogeneity among the included studies (*I*^2^ = 0%). Unsurprisingly, sensitivity analysis by excluding one study at a time showed consistent results, which further confirmed the robustness of the finding. Taken together, results of our meta-analysis confirmed that in adults following cardiac surgery, RIPC is not effective to reduce the incidence of POD. Currently, the mechanisms underlying the pathogenesis of POD remain largely unknown. It is generally accepted that multiple mechanisms may be involved in the pathogenesis of POD, such as inflammation, activated cytokines, and the neurochemical imbalances that affect neurotransmission (Oh & Park, 2019). Moreover, multiple risk factors have been identified underlying the development of POD, such as advanced age, preexisting cerebral and affective disorders, preoperative fluid fasting and dehydration, perioperative bleeding and hypovolemia, hyponatremia or hypernatremia, and the use of drugs with anticholinergic effects (Aldecoa et al., 2017). The physiological efficacy of RIPC is to meliorate the extent of ischemic-reperfusion injury, which may be simply not adequate to prevent multiple possible mechanisms that involved in the pathogenesis of POD (Pieri et al., 2020).

Similarly, six RCTs (Jing & Zheng, 2011; Joung et al., 2013; Meybohm et al., 2013; Hudetz et al., 2015; Meybohm et al., 2018; Gasparovic et al., 2019) were available for the meta-analysis evaluating the efficacy of RIPC on POCD after cardiac surgery. The sample sizes of the included RCTs were generally small, with a total of 659 patients observed and 245 with POCD. Pooled results showed that RIPC was not associated with significantly reduced POCD after cardiac surgery, although moderate heterogeneity was noticed. Sensitivity analysis by omitting one study at a time also showed consistent results, suggesting the robustness of the findings. However, it should be noticed that excluding the study by Hudetz et al. (Hudetz et al., 2015) substantially reduced the heterogeneity of the meta-analysis (*I*^2^ from 44 to 0%), suggesting that this study is the major source of heterogeneity. Interestingly, the study by Hudetz et al. is different from others in the diagnostic criteria for POCD. POCD was defined as a substantial decline of performance on ≥ 1 cognitive test in this study (Hudetz et al., 2015), while in the other studies, substantial declined performance on ≥ 2 cognitive test were requested. The relative loose criteria for the diagnosis of POCD in this study may lead to more patients diagnosed as POCD, and this is the only included RCT which showed that RIPC significantly reduced POCD after cardiac surgery. These findings may suggest that the effect of RIPC on POCD following cardiac surgery may be different according to the different diagnostic criteria for POCD applied among the included studies. However, in view of the emerged consensus regimens for neurocognitive testing and diagnostic criteria for POCD, such as the Recommendations for the Nomenclature of Cognitive Change associated with Anaesthesia and Surgery (2018) (Evered et al., 2018), studies evaluating the possible preventative strategies for POCD diagnosed with standardized criteria are needed. The result of this meta-analysis highlighted the important influence of definitions of POCD on the interpretation for studies that evaluated the potential preventative strategies for POCD (Needham et al., 2017).

The strengths of the current meta-analysis included rigorous literature search, strict inclusion and exclusion criteria, and performance of multiple sensitivity analysis to evaluate the potential source of heterogeneity. Besides, this study also has limitations. Firstly, as previously indicated, regimens for neurocognitive testing and diagnostic criteria for POCD varied among the included studies, and the difference in the definition of POCD may affect the results of the meta-analysis. Furthermore, we did not have access to the individual patient data. Accordingly, potential influences of patient or study characteristics on the outcomes of the meta-analysis could not be evaluated. Moreover, the sample sizes of the included RCTs varied significantly, particularly for the outcome of POD. The study with largest sample size (Meybohm et al., 2015) comprised over half of the included patients of the meta-analysis, which may primarily contribute to the overall results. Finally, the potential risk of publication bias was noticed for the outcome of POCD. However, further “trim-and-fill” analysis by incorporating the hypothesized studies with a positive result did not significantly change the overall results of the meta-analysis.

## Conclusion

In conclusion, results of this meta-analysis showed that RIPC does not significantly reduce the incidence of POD or POCD in adults following cardiac surgery. Although these findings may be validated in large-scale RCTs, particularly for the results of POCD, based on these findings, RIPC should not be routinely used as a preventative measure for POD and POCD in adult patients after cardiac surgery.

## Data Availability

The datasets used and/or analyzed during the current study are available from the corresponding author on reasonable request.

## Abbreviations

POCD: Postoperative cognitive dysfunction
POD: Postoperative delirium
CABG: Coronary artery bypass grafting
RIPC: Remote ischemic preconditioning
RCT: Randomized controlled trial
